# A Concave Nanogap for Ultrasensitive Aptamer-Based SERS Detection and *In Situ* Imaging of Heavy Metal Ions

**DOI:** 10.1021/acs.analchem.5c04843

**Published:** 2025-10-16

**Authors:** Ting Wang, Feiya Sheng, Sijia Wu, Yifei Mao, Juewen Liu, Peng Li, Jinchao Wei

**Affiliations:** † Macau Centre for Research and Development in Chinese Medicine, State Key Laboratory of Mechanism and Quality of Chinese Medicine, Institute of Chinese Medical Sciences, 59193 University of Macau, Macau 999078, China; ‡ School of Basic Medical Sciences, 74707 Chengdu University, Chengdu 610106, China; § Department of Chemistry, Waterloo Institute for Nanotechnology, 8430 University of Waterloo, Waterloo, Ontario N2L 3G1, Canada

## Abstract

Heavy metal pollution can lead to irreversible damage to human health. Surface-enhanced Raman scattering (SERS) platforms are versatile for toxicity monitoring and *in situ* imaging, but they are often limited by false positives. In this study, we proposed the use of concave-facet nanocubes (CF NCs) to create sufficiently large nanogaps, which can be used for the deposition of probes such as aptamers. Aptamers adopt an extended configuration within these nanogaps, effectively preventing compression-induced false signals. By utilizing the robust electromagnetic field present within the nanogaps, the developed SERS platform has demonstrated a broad detection range of 0.1 to 1000 nM for Hg^2+^ and a limit of detection as low as 0.1 nM. This platform was further applied to the analysis of Pb^2+^ and Cd^2+^. Furthermore, this system was applied to visualize the distribution of Hg^2+^ ions in zebrafish larvae, providing detailed insights into Hg^2+^ bioaccumulation. This work presents a universal strategy for concave-shaped nanoparticle-assisted SERS detection of analytes with weak or no Raman signatures, such as heavy metal ions, while reducing false-positive results in analysis. It also underscores the promising potential for targeted *in situ* imaging in biological samples, serving as a tool for investigating the mechanisms of toxicity in various substances.

## Graphical abstract

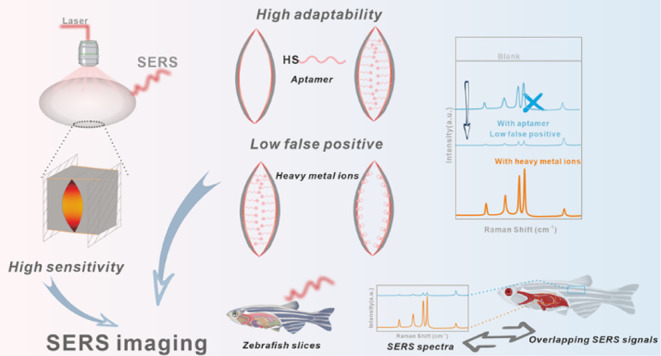

## Introduction

The homeostasis of metal ions is critical for maintaining human health, while disruptions to this balance can result in severe consequences, even for essential metal elements.^1^ Heavy metals bioaccumulate within organisms and biomagnify through the food chain, ultimately entering the human body and disrupting metal homeostasis, often leading to irreversible health damage. To address these risks, it is crucial to develop analytical platforms capable of timely, accurate, and rapid in situ detection of heavy metal content in biological systems.^2^ *In situ* bioimaging can reveal many mechanisms of metal transport in biological systems and their potential impact on metal toxicity.^3^ Existing techniques, such as inductively coupled plasma mass spectrometry (ICP-MS), fluorescence spectroscopy, electrochemical sensors, and colorimetry, have been widely employed in detecting trace heavy metals in the environment, achieving significant progress.^4^ Notably, some advanced colorimetric and fluorescent sensors can directly visualize heavy metal ions, allowing faster access to critical information.^5^ While several sensors are now available, their applications remain limited. Whether in relatively simple water environments or complex biological matrices, these sensors predominantly support single-channel detection or imaging, posing significant challenges for multiplexed analysis.

Surface-enhanced Raman scattering (SERS) has received sustained attention due to its unique fingerprint spectrum, nondestructive analysis, high sensitivity, and negligible autofluorescence. Consequently, SERS technology exhibits tremendous advantages and potential for ultrasensitive multiplex detection and in situ, nondestructive imaging.^6^ Recently, some studies have directly visualized targets in cells using SERS.^7,8^ Recent progress has shown that SERS-based probes not only enable the sensitive identification of metal ions in solution but also allow real-time visualization of biomolecules and toxic ions inside living cells. These developments highlight the potential of SERS for bridging fundamental detection with practical visualization, thereby enhancing its relevance in environmental monitoring and biomedical applications. However, obtaining fingerprint spectra remains highly challenging for heavy metal ions, small molecules with inherently low Raman cross-sections, and macromolecules such as proteins and nucleic acids. These difficulties are further compounded in biological samples, where the complex matrix frequently occupies the available hotspots.^9^ To address this limitation, researchers have turned to indirect detection strategies by utilizing reactions between target analytes and Raman-active reporter molecules or by employing functionalized aptamers labeled with reporter molecules to indicate analyte concentrations.^10–12^ Although this labeling approach provides a convenient solution to overcome these challenges, it also raises the issue of false positives caused by spatial misalignment, thereby undermining the reliability of SERS-based detection.

Concave nanoparticles have garnered significant attention in various fields due to their exceptional performance in electrocatalysis,^13,14^ biomarker detection,^15^ and disease therapy.^16^ Compared to conventional nanoparticles, concave nanoparticles exhibit weaker shape complementarity between adjacent particles, which reduces the overlap of surface-bound ligands such as DNA.^17^ As mentioned above, one underlying cause of false-positive signals in SERS is the misplacement of reporter molecules into adjacent hotspots on the nanoparticle surface. Theoretically, concave nanoparticles can mitigate this issue, thereby decreasing the probability of false positives. Furthermore, studies have demonstrated that concave noble-metal nanoparticles as SERS substrates exhibit higher enhancement factors compared to their nonconcave counterparts. This superior performance highlights the untapped potential of concave noble-metal nanoparticles in visualizing the distribution of targets within tissues, cells, or other biological samples. In this work, we utilized concave facet Au/4-MB@Ag nanocubes (CF Au/4-MB@Ag NCs) for the detection of mercury ions (Hg^2+^) as a model analyte to validate the above concept. Aptamers are excellent for metal sensing because of their high stability, ease of labeling, and programmable structures.^18–20^ A DNA aptamer,^21^ rich in T bases and modified with Bodipy R6G as a Raman reporter, served as a probe for Hg^2+^ detection, indirectly reflecting its concentration. The SERS signal of the blank solution was weak and uniform, while the SERS intensity demonstrated a positive correlation with Hg^2+^ concentration over a wide dynamic range due to the unique concave structure, with clear signals down to 0.1 nM. This sensor was successfully applied to detect Hg^2+^ in water and herbal plants. More importantly, uptake and colocalization of Hg^2+^ in zebrafish by using this platform were achieved. Furthermore, this strategy was extended to detect two additional heavy metal ions: lead (Pb^2+^) and cadmium (Cd^2+^). This work demonstrates for the first time the potential of concave-shaped nanoparticles for SERS detection of analytes that have weak or no Raman signatures, and even *in situ* imaging of targets in real biological samples.

## Experimental Section

### Synthesis of CF Au/4-MB@Ag NCs

#### Synthesis of Au/4-MB@Ag NCs with Controlled Size

Au NPs were synthesized by sodium borohydride reduction, followed by mixing them with different concentrations of 4-MB solution and incubating them for 1 h. 13 mL of Au@4-MB NPs were added to an 18 mM CTAC solution, heated at 65 °C for 20 min in a water bath, and then different amounts of 2 mM AgNO_3_ and 50 mM AA solution were added simultaneously and stirred for 3 h.

#### Synthesis of CF Au/4-MB@Ag NCs

1 mL portion of 0.1 mM HAuCl_4_ was added dropwise to a mixed solution consisting of Au/4-MB@Ag NCs solution, 100 mM CTAC, 100 mM AA, and NaOH. The pH of the solution was 11. The mixture was then centrifuged and washed 3 times immediately after the addition to obtain CF Au/4-MB@Ag NCs precipitate.

#### Synthesis of CF Au/4-MB@Ag NCs-Aptamer

Aptamers were dissolved in a Tris-HCl buffer (pH 7.4) to prepare stock solutions. For thiol activation, 50 μL of 10 mM TCEP was incubated with 50 μL of 10 μM aptamer for 1 h at room temperature in the dark. The precipitated CF Au/4-MB@Ag NCs were then mixed with the activated aptamer, followed by the immediate addition of 190 μL of 0.01 wt % SDS. The mixture was incubated overnight at 37 °C under gentle shaking. The resulting complexes were washed twice and concentrated 5-fold to obtain the final SERS substrate.

#### Sample Preparation and Test

All cation ion solutions were first prepared at specific concentrations as presolutions. The subsequent procedures were similar to our group’s previous work. Briefly, the prepared solution was mixed with CF Au/4-MB@Ag NCs, incubated for 30 min, dropped onto a silicon wafer, dried, and then analyzed using a Raman spectrometer to obtain SERS spectra. For zebrafish slices, 2 mpf zebrafish were incubated with a Hg^2+^ standard solution, followed by fixation in formalin solution for 24 h. The samples were then cryosectioned into 20 μm-thick slices. CF Au/4-MB@Ag NCs solution was directly applied to the zebrafish slices, dried at room temperature, and scanned using a Raman spectrometer to record the corresponding SERS spectra. All SERS spectra were collected using an excitation wavelength of 633 nm, a power density of 1%, a 50× objective lens, and an exposure duration of 10 s. All spectra were baseline-subtracted unless indicated otherwise.

## Results and Discussion

### Fabrication and Characterization of the CF Au/4-MB@Ag NCs

The method for synthesizing concave-facet Au@4-MB@Ag nanocubes (CF Au/4-MB@Ag NCs) was based on our previous work.^22^ First, core–shell Au/4-MB@Ag NCs were synthesized via a seed-growth approach and subsequently employed as sacrificial templates. The galvanic replacement and coreduction reactions under alkaline conditions facilitated the formation of concave-faceted structures, as shown in Figure 1a. However, encoding internal standard (IS) molecules (mercaptobiphenylcarbonitrile, 4-MB) onto the core NPs significantly influenced the morphology of the subsequent shell layers. As illustrated in Figure S1, the pristine Au core NPs serve as the foundation, while Figure S2 demonstrates the morphological variations in the shell layers upon introducing IS at different concentrations. Notably, the impact on shell morphology was negligible when the concentration of IS was below 1 × 10^–7^ M. Additionally, shell thickness emerged as a critical factor in determining both NP morphology and SERS enhancement performance. TEM images (Figures 1b and S3) reveal that CF Au/4-MB@Ag NCs with varying shell thicknesses were synthesized by using Au/4-MB@Ag NCs as sacrificial templates in a reaction solvent containing CTAC, AA, and NaOH at pH 11. The zeta potential of the CF Au/4-MB@Ag NCs was approximately +50 mV, which can be attributed to the presence of CTAC on the CF Au/4-MB@Ag NCs’ surface (Figure S4). Figure S5 shows that the XRD pattern of CF Au/4-MB@Ag NCs is very similar to Au and Ag standard XRD patterns, which indicates that the face-centered cubic (fcc) CF Au/4-MB@Ag NCs were formed due to the strong peaks of (111), (200), and (220). The shell thickness (***S***_***T***_) ranged from 1 to 11 nm, and the concave facets were formed as a result of the galvanic replacement and coreduction reactions. It is worth noting that TEM images of the 11 nm CF Au/4-MB@Ag NCs show some noncubic NPs (Figure S3), a phenomenon attributable to the challenges in morphology control associated with excessive precursor concentrations. The degree of concavity in typical CF Au/4-MB@Ag NCs was quantified by measuring the horizontal distance from the vertex to the lowest point of the concave facet, which was approximately 5 nm. Importantly, the presence of a substantial number of CTAC bilayer molecules on the NP surface slightly inhibited the proximity between individual NPs, as illustrated in Figure S6. This observation indicates that our proposed nanoparticle aggregates maintain an interparticle distance of approximately 10 nm, providing sufficient space for the placement of aptamers.

**Figure 1:**
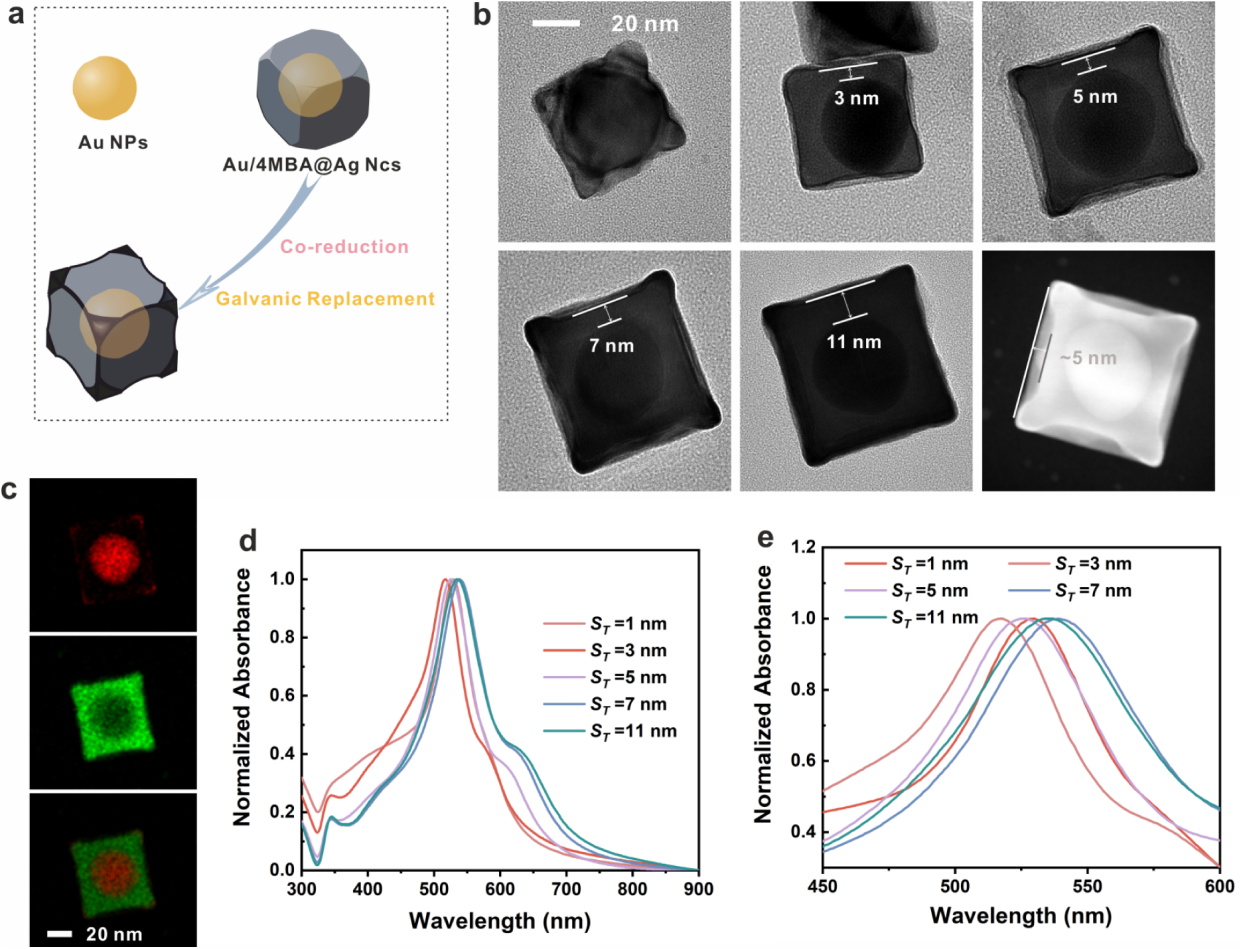
(a) Schematic diagram of the synthesis of CF Au/4-MB@Ag NCs. (b) Typical TEM images of the CF Au/4-MB@Ag NCs with different thicknesses; the vertical distance from the lowest point of the curved surface to the plane was taken as a measure of the degree of concavity degree. The distance between CF Au/4-MB@Ag NCs is nearly 5 nm. (c) Typical STEM-EDS elemental mapping results of CF Au/4-MB@Ag NCs. (d) UV–vis absorption spectra of the CF Au/4-MB@Ag NCs with different thicknesses. (e) Enlarged view of the absorption spectra.

The successful synthesis of CF Au/4-MB@Ag NCs was further validated by STEM-EDS elemental mapping (Figure 1c), which revealed that Au was primarily distributed within the core and on the {111} and {110} facets, while Ag was present on the {100}, {110}, and {111} facets. STEM-EDS elemental analysis indicated that Au, Ag, and Cu collectively accounted for over 90% of the total elemental composition, with the proportion of Ag increasing with shell thickness (Figure S7). The presence of Cu was attributed to the use of copper grids for TEM imaging. Furthermore, detailed UV–vis spectra of CF Au/4-MB@Ag NCs with varying shell thicknesses are presented in Figure 1d,e. While CF Au/4-MB@Ag NCs with ***S***_***T***_ = 1 nm exhibited four prominent vertices, higher-order plasmon modes at 340 and 388 nm were absent, likely due to the excessively thin Ag shell. As ***S***_***T***_ increased, these higher-order plasmon modes became discernible, accompanied by a red shift of the absorption peak. Interestingly, the absorption peak of CF Au/4-MB@Ag NCs with ***S***_***T***_ = 11 nm deviated from the expected trend, lying between those of ***S***_***T***_ = 5 nm and ***S***_***T***_ = 7 nm. This anomaly can be attributed to the complex morphology of CF Au/4-MB@Ag NCs with ***S***_***T***_ = 11 nm, corroborating the TEM observations. Figure 1e provides an enlarged view of the spectral variations, as shown in Figure 1d. In summary, these characterization results collectively confirm the successful and controllable synthesis of CF Au/4-MB@Ag NCs, offering valuable insights into their structural and optical properties.

### The SERS Performance of the CF Au/4-MB@Ag NCs

#### The SERS Reliability of the CF Au/4-MB@Ag NCs

The SERS performance of CF Au/4-MB@Ag NCs was evaluated with a focus on two critical aspects: signal reliability and sensitivity.^23^ As mentioned above, the morphology of the NPs remains unaffected by IS concentrations below 1 × 10^–7^ M. However, a stable IS signal is crucial for precise quantitative SERS detection, as it significantly enhances reliability and will be discussed in detail in subsequent sections. The IS signals and corresponding SERS spectra of NPs synthesized with varying IS concentrations are summarized in Figures S8 and S9. The SERS signals of NPs with IS concentrations below 1 × 10^–8^ M were indistinguishable, leading to the selection of 5 × 10^–8^ M as the optimal IS concentration for subsequent CF Au/4-MB@Ag NC synthesis.

The CF Au/4-MB@Ag NCs feature four inwardly concave facets, and the self-assembly between these NCs generates numerous nanogaps (∼10 nm). The size of these nanogaps can be fine-tuned by adjusting the relative rates of the two reactions.^22^ These nanogaps not only induce strong localized electromagnetic fields (EMFs) (i.e., hotspots) but also crucially facilitate the entry and deposition of macromolecules, thereby enhancing the versatility and sensitivity of CF Au/4-MB@Ag NCs.^24^ To validate this concept, we employed a thymine-rich aptamer (∼8 nm when stretched), a sequence known for selective binding to Hg^2+^ ions. The 3′-terminal end of the aptamer was modified with a sulfhydryl group to conjugate to the CF Au/4-MB@Ag NC surfaces, while the 5′-terminal end was tagged with the Raman reporter. The strong affinity between Hg^2+^ and T bases enables this aptamer to selectively capture Hg^2+^, forming the “T-Hg-T” structure.^25^ The Hg^2+^-aptamer folded back upon encountering Hg^2+^, thereby shortening the distance between the Raman reporter molecule and the CF Au/4-MB@Ag NC surface. Raman reporter molecules near the surface are in an enhanced EMF, consequently amplifying the Raman signal of the reporter molecule, as depicted in Figure 2a. The corresponding SEM image of the substrate is shown in Figure S10. Bodipy R6G served as a reporter molecule to record the SERS spectra of aptamers in various states, with the 1511 cm^–1^ peak being utilized as a quantitative peak. Figure 2b illustrates the SERS spectrum of the CF Au/4-MB@Ag NCs-aptamer with and without Hg^2+^. The characteristic peak at 1511 cm^–1^ exhibits weak intensity in the absence of Hg^2+^ due to the weak EMF near the Bodipy R6G molecule. However, its intensity substantially escalates upon the addition of Hg^2+^. This rapid intensity augmentation confirms the formation of the “T-Hg-T” structure between Hg^2+^ and T bases, folding the linear aptamer back into a curved “double strand”. To evaluate the potential for reducing false positives, the SERS signals of CF Au/4-MB@Ag NCs-aptamer conjugates without Hg^2+^ were measured across varying shell thicknesses, and the relative intensities are summarized in Figure 2c. Regardless of the shell thickness, all NPs exhibited low and stable signals in the absence of Hg^2+^. Notably, NPs with ***S***_***T***_ = 1 nm failed to provide reliable data due to the absence or insufficient thickness of the Ag shell, which led to the loss of IS signals. Additionally, 15 random points on NPs with ***S***_***T***_ = 7 nm were tested, revealing consistent trends in the characteristic peaks and IS intensities. This excellent consistency resulted in near-identical intensity ratios, elucidating the mechanism by which the incorporation of the IS improves the reliability of the SERS detection system (Figure 2c). To further support our hypothesis, we randomly selected 40 points from blank samples of the CF Au/4-MB@Ag NCs-aptamer and Au/4-MB@Ag NCs-aptamer and calculated the corresponding RSD values (Figure S11). Notably, the RSD decreased sharply from 32.4% to 13.8% upon the introduction of the concave-facet configuration. In addition, the relative ratio of Au/4-MB@Ag NCs-aptamer was much higher than that of CF Au/4-MB@Ag NCs-aptamer, indicating that Bodipy R6G was positioned closer to the surface of the plasmonic nanoparticles. Collectively, these findings confirm that the proposed concave-facet nanoparticles can effectively reduce the likelihood of false positives.

**Figure 2:**
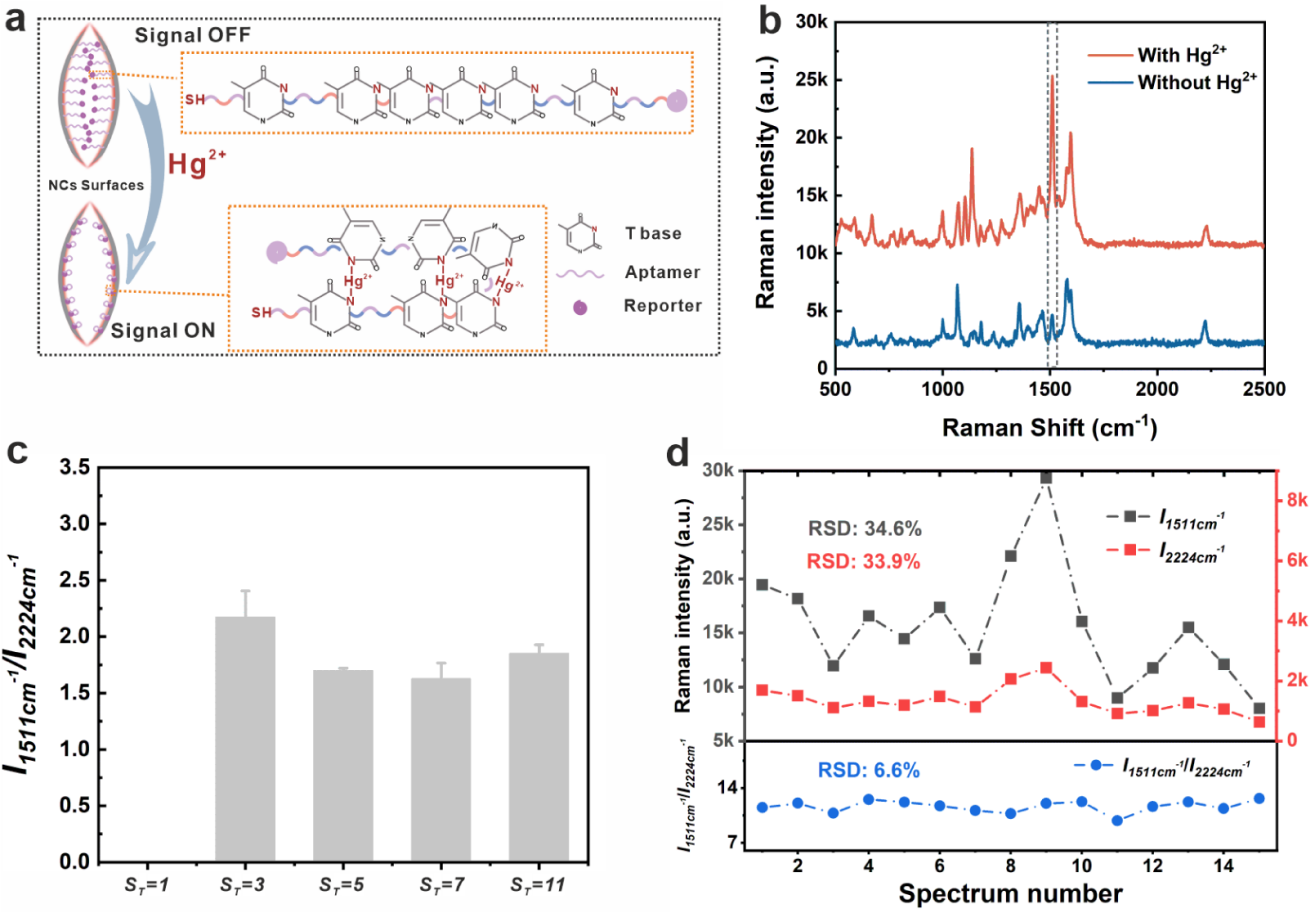
(a) Schematic diagram of the mechanism between Hg^2+^ and the aptamer. (b) SERS spectrum of CF Au/4-MB@Ag NCs-aptamer with and without Hg^2+^. (c) Relative SERS intensities of the quantitative peak (1511 cm^–1^) and IS peak (2224 cm^–1^) in the blank sample with different thicknesses of CF Au/4-MB@Ag NCs. (d) SERS intensities of the quantitative peak and IS peak, as well as their intensity ratios, across the randomly selected 15 spots.

In conclusion, the concave-facet NPs significantly reduce the likelihood of false positives, while the embedded IS enhances the uniformity of the SERS signals. These features endow our NPs with high reliability, particularly for macromolecule-assisted SERS detection systems.

#### The SERS Sensitivity of the CF Au/4-MB@Ag NCs

Although CF Au/4-MB@Ag NCs with a curved facet configuration were prepared by controlling two chemical reactions, these NCs fundamentally remain core–shell NPs. Consequently, the thickness of the shell significantly impacts their SERS performance. 1 μM Hg^2+^ was used as an evaluation sample throughout the experiment unless otherwise specified. Figure 3a presents the intensity of the IS peak, the quantitative peak, and their ratios at each shell thickness to observe the relationship more intuitively between shell thickness and SERS performance. Similar to the above, the CF Au/4-MB@Ag NCs-aptamer with ***S***_***T***_ = 1 nm shows losses of the IS peak at 2224 cm^–1^. This occurred partially because the concentration of the IS was only 5 × 10^–8^ M, which is extremely low to strictly control the morphology of CF Au/4-MB@Ag NCs. Additionally, the SERS intensity of 4-MB is influenced by both the Au core and the Ag shell, and the insufficient Ag shell reduces the SERS intensity. The Ag shell also affects the SERS signal intensity of Bodipy R6G. While the intensity of the characteristic peak increases with the gradual thickening of the Ag shell, it suddenly drops at ***S***_***T***_ = 11 nm. This drop is attributed to the excessively thick Ag shell blocking the laser penetration and the diverse configurations of the CF Au/4-MB@Ag NCs. Figure 3b shows the corresponding SERS spectrum, and the intensity trends of other Raman peaks of Bodipy R6G align with the quantitative peak. From this perspective, ***S***_***T***_ = 7 nm appears to be optimal, as it provides both a stable IS signal and a sufficiently high SERS characteristic peak signal. Such statements are also supported by the simulation results in Figure 1c and Figure S12. Therefore, the CF Au/4-MB@Ag NC with ***S***_***T***_ = 7 nm was selected for further study.

**Figure 3:**
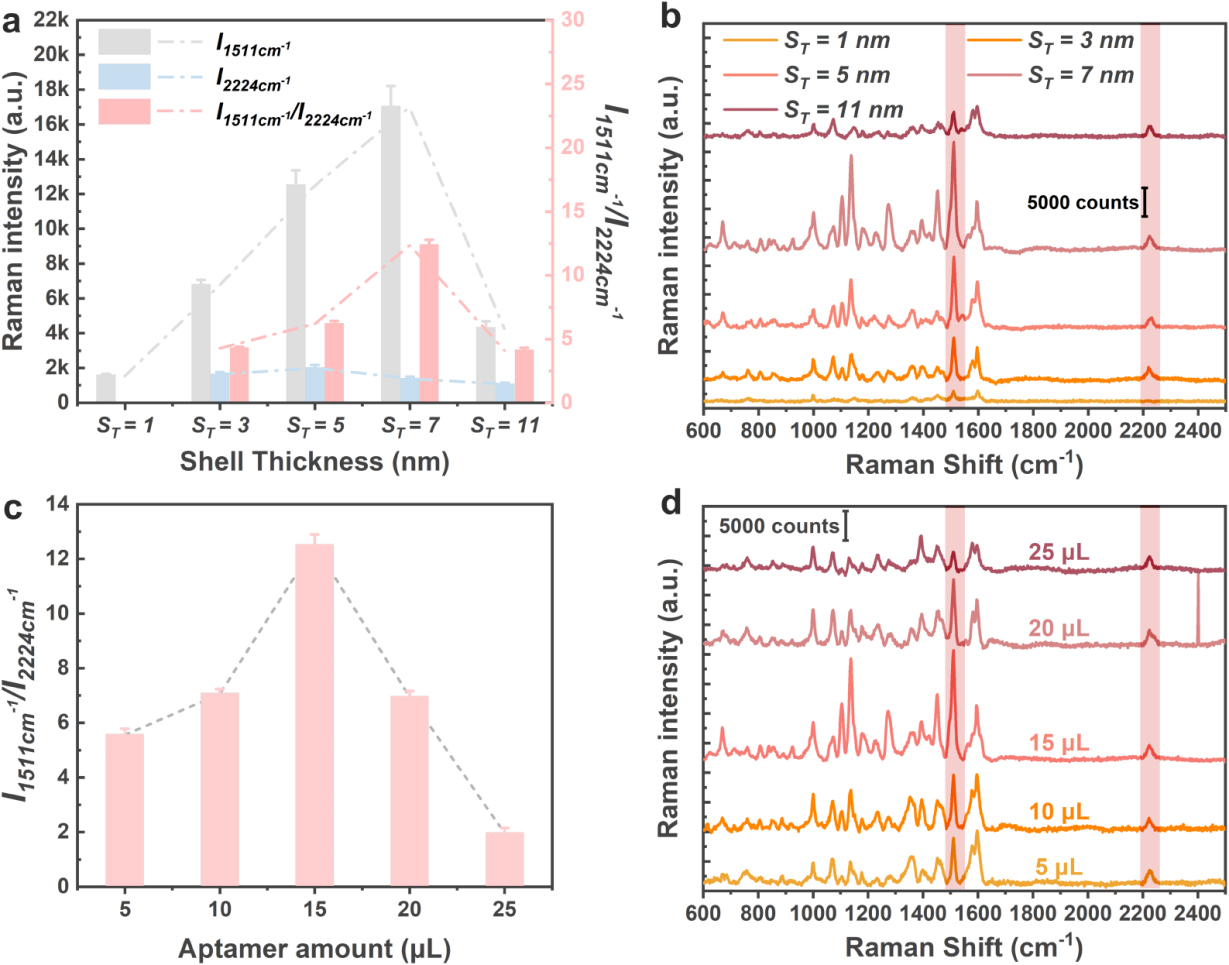
(a) Histograms of SERS intensities for the quantitative peak, IS peak, and their ratio. (b) The typical corresponding SERS spectrum of (a). (c) Relative SERS intensities of the quantitative and IS peaks when CF Au/4-MB@Ag NCs were incubated with different amounts of aptamer. (d) The typical corresponding SERS spectrum of (c).

It is important to note that Bodipy R6G is a fluorescent molecule, and excessive fluorescence from Bodipy R6G molecules can inhibit its Raman signal, reduce the SERS performance, and raise the baseline, as shown in Figure S13. Thus, the relationship between the amount of Hg^2+^-aptamer and the SERS performance was systematically evaluated. Figure 3c presents the intensity changes of the quantitative peak with an additional amount of aptamer. The SERS signal intensity increases with the addition of aptamer but decreases when more than 15 μL is added. The corresponding SERS spectrum is shown in Figure 3d. Overall, the size of CF Au/4-MB@Ag NCs and the amount of aptamer significantly affect the SERS performance and detection ability. Ultimately, we chose to add 15 μL of aptamer and incubate it with CF Au/4-MB@Ag NCs with ***S***_***T***_ = 7 nm to prepare the CF Au/4-MB@Ag NCs-aptamer sensor, which serves as the substrate for subsequent Hg^2+^ detection.

#### Reproducibility and Detection Capacity of the CF Au/4-MB@Ag NCs-Aptamer Sensor

To quantitatively evaluate SERS intensities, the RSD value of SERS signals was recorded at multiple randomly selected points or local areas (Table S1). Samples prepared using the traditional drip-dry method often exhibit the coffee ring effect, which concentrates NPs and target molecules, thereby reducing the limit of detection (LOD). However, ring-shaped samples make it difficult to collect sufficient SERS signals from a large and regular area. In this work, we overcame this limitation by scanning the entire coffee ring with a step size of 25 μm, as shown in Figure S14, obtaining 611 spectra. Figure 4a,b further illustrates the mapping results of the SERS single peak intensity (*I*_1511 cm–1_) and SERS relative intensity (*I*_1511 cm–1_/*I*_2224 cm–1_). The RSD value of the SERS single peak intensity is as high as 50.9%. Such a high RSD is attributed to the tendency of the CF Au/4-MB@Ag NCs-aptamer to deposit disorderly on the contact line of colloidal droplets, causing reporter molecules to be in chaotic “hotspots” and resulting in fluctuating SERS single signals. Since the IS peak is utilized to calibrate the fluctuating signals in the CF Au/4-MB@Ag NCs-aptamer, the RSD of the relative SERS intensity mapping result significantly lowered to 9.8%. Despite the unpredictable distribution of the CF Au/4-MB@Ag NCs-aptamer, the IS signal fluctuation aligns with the characteristic peak fluctuation trend, reducing the RSD of relative intensity. These results demonstrate the high uniformity of our samples, which greatly contributes to the improvement of the SERS signal reproducibility. Reproducibility between batches is another critical issue hindering the advancement of SERS in accurate trace detection. To evaluate batch bias, the SERS spectra from 10 batches with 5 randomly selected points per batch were examined. Figure 4c summarizes the relative intensities of quantitative and IS peaks across multiple batches. The low RSD of 6.5% indicates excellent reproducibility, confirming the application potential of CF Au/4-MB@Ag NCs-aptamer in SERS trace detection.

**Figure 4:**
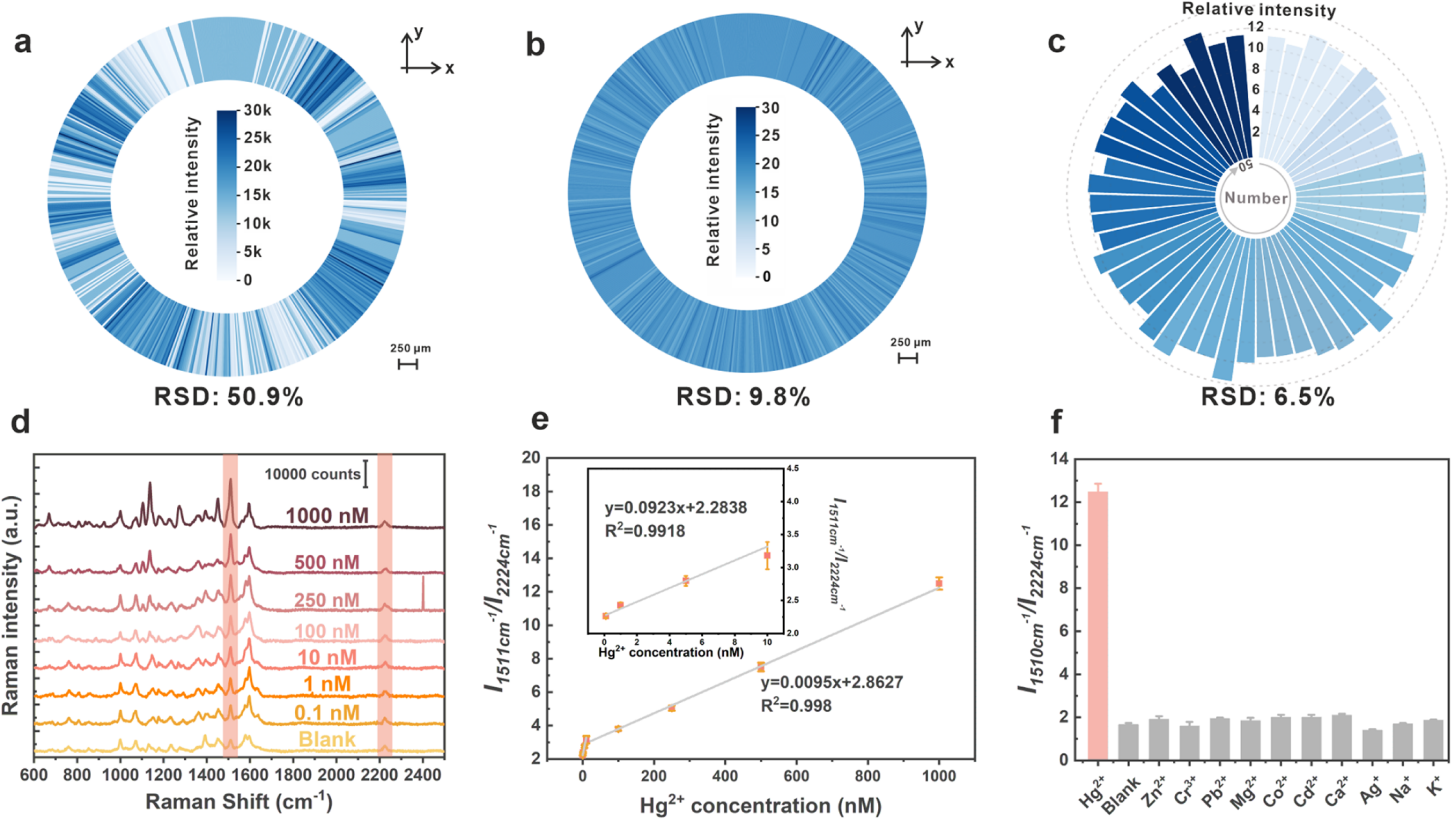
(a, b) The mapping results of SERS signals of CF Au/4-MB@Ag NCs (a) and the relative SERS signal of CF Au/4-MB@Ag NCs (b) on the circle edge. (c) Relative SERS intensities of the quantitative peak across 10 batches based on the CF Au/4-MB@Ag NCs. (d) Typical SERS spectra of CF Au/4-MB@Ag NCs-aptamer with different Hg^2+^ concentrations. (e) Relationships between the relative SERS intensity of the quantitative peak and Hg^2+^ concentration. (f) Relative SERS intensities of Hg^2+^ and other cation ions with CF Au/4-MB@Ag NCs-aptamer.

Based on the above excellent evaluation of our proposed CF Au/4-MB@Ag NCs-aptamer system, it has great potential to be used for quantitative detection. Figure 4d further shows the SERS spectral changes caused by Hg^2+^ in Bodipy R6G molecules, with concentrations ranging from 0.1 nM to 1 μM. Unlike the gradually increasing Bodipy R6G characteristic peaks, the IS peak intensity of CF Au/4-MB@Ag NCs-aptamer remains relatively constant, which allows the relative intensity of the quantitative peak to quantify Hg^2+^ concentration. The relative intensity of the quantitative peak at different Hg^2+^ concentrations is calculated, as shown in Figure 4e, and demonstrates a strong linear relationship (*R* ^2^ > 0.99). In the 1 μM–10 nM range, the regression formula is *y* = 0.0095*x* + 2.8627, while in the 10–0.1 nM range, it is *y* = 0.0923*x* + 2.2838. Despite the different linear formulas for high and low Hg^2+^ concentrations, there is a clear SERS signal for Hg^2+^ concentrations as low as 0.1 nM. The curved facets of CF Au/4-MB@Ag NCs fabricate the strong EMF and provide ample space for macromolecules, such as aptamers, to enter the “hotspot”. Therefore, the LOD of our system is competitive with traditional core–shell-based SERS substrates (Table S2).

To confirm the specificity of the 1511 cm^–1^ characteristic peak response to Hg^2+^, other metal ions at 10 μM were tested. All of these ions showed responses similar to the blank sample (ultrapure water), which were much lower than the enhancement caused by 1 μM Hg^2+^ (Figure 4f). However, Cu^2+^ exhibited a different SERS spectrum and eliminated all SERS signals, including the IS signal (Figure S15). This phenomenon may be attributed to the remaining bilayer CTAC molecules on the surface of CF Au/4-MB@Ag NCs-aptamer, which can react with Cu^2+^ and then etch the Ag shell into Ag^+^.^26^ The lack of an Ag shell decreases the IS and characteristic peak SERS intensities, thereby destroying the detection capability. To address this, adding a metal chelator such as EDTA to bind Cu^2+^ can prevent its reaction with the Ag shell and restore the detection capability. As shown in Figure S16, EDTA-Cu^2+^ and EDTA-Hg^2+^ complexes were added to the CF Au/4-MB@Ag NCs-aptamer to monitor the IS SERS signals. Interestingly, this SERS spectrum contains the IS peak and various signal peaks due to the EDTA.^27^ This suggests that using specific Cu^2+^ chelators could block the reaction between Cu^2+^ and the Ag shell, restoring the detection ability. This approach will be explored in future work. In summary, these results highlight the great potential of our proposed CF Au/4-MB@Ag NCs-aptamer sensor for accurate quantitative detection in practical applications.

### The Broad Adaptability of the CF Au/4-MB@Ag NCs

As mentioned before, our proposed CF Au/4-MB@Ag NCs equipped with IS and curved facets offer high reliability, sensitivity, and ample space, making them particularly suitable for macromolecule or macromolecule-assisted SERS detection. To confirm the broad adaptability of the CF Au/4-MB@Ag NCs, an aptamer specific for cadmium ions (Cd^2+^) and a DNAzyme specific for lead ions (Pb^2+^) were also employed for trace detection. Similar to the Hg^2+^ aptamer, one end of the single-stranded DNA is labeled with a Raman reporter, and the other end is modified with a sulfhydryl group, as shown in Figure 5a. The Cd^2+^ aptamer was first reported by the Pei group.^28^ The Cd^2+^ recognition unit is located in the middle of the Cd^2+^-aptamer, which surrounds Cd^2+^ after specific recognition. Subsequently, the straight single-stranded aptamers bend inward and bring the Raman reporter molecule closer to the CF Au/4-MB@Ag NC surface, as shown in Figure 5a–I. The inward-folded aptamers position the Raman reporter molecules in a stronger EMF and enhance the Raman signals, thereby suiting Cd^2+^ SERS trace detection.

**Figure 5:**
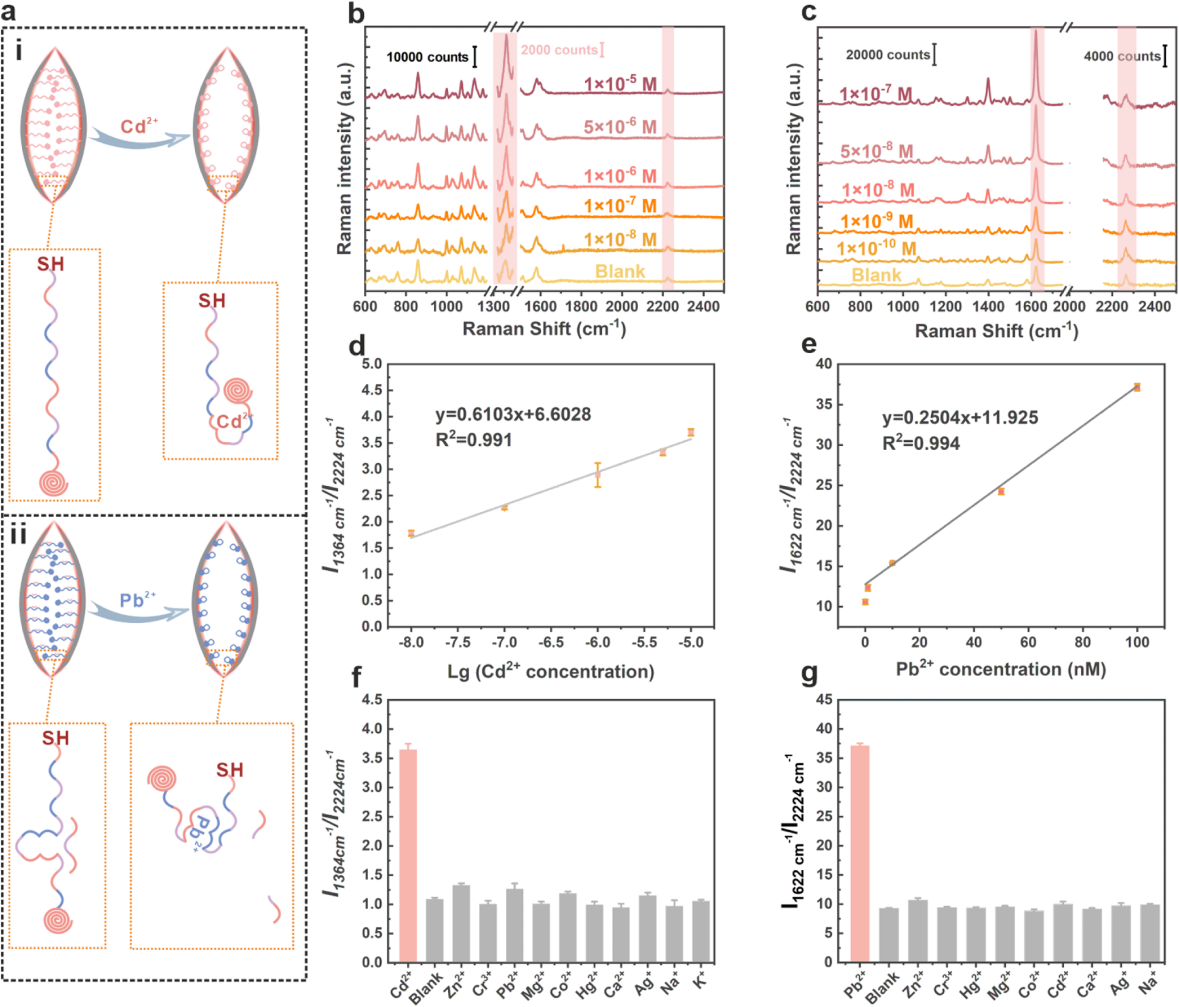
(a) Schematic diagram of the mechanism between Hg^2+^ and the capture unit. (b, c) Typical SERS spectra of CF Au/4-MB@Ag NCs-aptamer with different Cd^2+^ (b) and Pb^2+^ (c) concentrations. (d, e) Relationships between the relative SERS intensity of the quantitative peak and Cd^2+^ (d) and Pb^2+^ (e) concentrations. (f, g) Relative SERS intensities of Cd^2+^ (f) and Pb^2+^ (g) and other cation ions with CF Au/4-MB@Ag NCs-aptamer.

To detect Pb^2+^ ions, we employed a Pb^2+^-specific DNAzyme named 17E.^29^ As shown in Figure 5a-II, the DNAzyme that specifically recognizes Pb^2+^ consists of a long enzyme strand and a short substrate strand. Simultaneously, multiple A bases at the 5′ end of the long DNA strand allow the reporter molecules to approach the surface of CF Au/4-MB@Ag NCs due to the strong affinity between the A base and the Au and Ag metal NPs. Cy5 and methyl blue molecules served as Raman signal reporter molecules for Cd^2+^ and Pb^2+^, respectively. Figure 5b,c reveal the SERS spectra of different concentrations of Cd^2+^ and Pb^2+^ in the CF Au/4-MB@Ag NCs-macromolecule system. Similar to Hg^2+^, the SERS signal intensity of Cy5 and methyl blue gradually increases as the concentration of Cd^2+^ and Pb^2+^ increases, while the IS signal remains stable. This indicates that the CF Au/4-MB@Ag NCs-macromolecule system has great potential for the accurate quantitative detection of Cd^2+^ and Pb^2+^ by using the relative intensity of the quantitative peak. Using 1362 cm^–1^ and 1610 cm^–1^ as the quantitative peaks for Cd^2+^ and Pb^2+^ due to their high peak intensity, the ratio of the quantitative peak to the IS peak was calculated. The concentrations of Cd^2+^ and Pb^2+^ show a linear relationship with the relative intensity of the quantitative peak, as illustrated in Figure 5d,e. However, the detection range of Cd^2+^ is 10 μM to 10 nM, which is higher than Hg^2+^, since the recognition site of the Cd^2+^ aptamer is far from the 3′-terminal end of the aptamer. Despite the higher detection concentration for Cd^2+^, the relative intensity of the quantitative peak and Cd^2+^concentration exhibit an excellent linear relationship (*R* ^2^ > 0.99). At the same time, the detection range of Pb^2+^ is 0.1 nM to 100 nM, which is similar to that of Hg^2+^ and the *R* ^2^ of the regression curve between Pb^2+^ concentration and the relative intensity of the quantitative peak is also over 0.99.

The specificity of the CF Au/4-MB@Ag NC-macromolecule system was further verified by recording the SERS spectra of other metal cations and calculating the relative intensity of their quantitative peaks relative to the IS peak. As shown in Figure 5f,g, 10 μM and 100 μM metal cations were added in Pb^2+^ and Cd^2+^ systems to verify the selectivity. Although other metal cations caused slight signal fluctuations, the relative intensity of the quantitative peak was similar to that of the blank sample, confirming the specificity of the CF Au/4-MB@Ag NC-macromolecule system. It is worth noting that the SERS spectrum of Cu^2+^ still lacks an effective characteristic peak and IS peak for quantification. This phenomenon, in turn, reaffirms that Cu^2+^ destroys CF Au/4-MB@Ag NCs rather than the specific macromolecules. Overall, these results demonstrate that our proposed CF Au/4-MB@Ag NCs exhibit high sensitivity, reliability, and broad application potential, making them especially suitable for macromolecular substances or macromolecule-assisted SERS detection.

### Real Applications of the CF Au/4-MB@Ag NCs

Heavy metals are prevalent in the environment and often enter the human body through the digestive tract, leading to the accumulation of heavy metal ions and posing significant health risks. Therefore, accurately and sensitively detecting residual amounts of heavy metals in real samples is crucial. However, the complexity of real sample matrices, which contain various interfering components, presents a challenge for accurate SERS detection. Our proposed CF-Au/4-MB@Ag NCs are able to meet this challenge due to their high sensitivity, reliability, and broad adaptability, allowing them to accurately detect heavy metal residues in real samples. Hg^2+^ was systematically evaluated as a representative heavy metal ion. Due to the long residence time of mercury vapor in the atmosphere and its oxidation to soluble inorganic Hg^2+^, large quantities of water and soil can become contaminated. Consequently, heavy metal residues in fish, tap water, and plants, which rely on water and soil for survival, are of particular concern.^30^

#### *In Situ* Imaging of Heavy Metal Ions in Zebrafish

Zebrafish were utilized as a representative sample to systematically describe the accumulation and distribution of Hg^2+^. Briefly, zebrafish larvae at 2 months post-fertilization (mpf) were soaked in a 10 μM Hg^2+^ solution for 2 h and then sliced for analysis. Detailed procedures are described in the Materials and Methods section. Our proposed CF Au/4-MB@Ag NCs, with or without Hg^2+^ aptamers, were sprinkled on the slices, dried, and subjected to SERS detection with a 25 μm step size. The SERS spectrum of the whole zebrafish was recorded, and the relative intensity of the quantitative peak (1511 cm^–1^) to the IS peak (2222 cm^–1^) was calculated. Figure 6 presents the SERS analysis of Hg^2+^ in zebrafish. Figure 6a–c corresponds to Hg^2+^-zebrafish exposed to CF Au/4-MB@Ag NCs alone (6a), blank zebrafish, and Hg^2+^-zebrafish treated with the aptamer-modified CF Au/4-MB@Ag NCs-Hg^2+^ SERS substrate (**6b** and **6c**). Figure 6 (i–iii) shows the optical images of zebrafish slices under the Raman microscope, SERS signal distribution, and corresponding merged images, respectively. Almost no signal is observed in Figure 6a due to the absence of the Hg^2+^-aptamer. Although the proposed substrate reduces false positives, sporadic signals are visible in Figure 6b, likely resulting from the heterogeneous substrate distribution and the complex zebrafish matrix. In contrast, Figure 6c exhibits markedly stronger and more localized SERS signals. While the IS peak intensity fluctuates within a narrow range, the characteristic Hg^2+^ peaks show larger variations, indicating that the signal heterogeneity mainly reflects differences in local Hg^2+^ concentration rather than substrate distribution. To highlight the accumulation sites, the SERS distribution (Figure 6c (ii)) was overlaid with the optical image (Figure 6c (i)) to produce Figure 6c (iii), clearly revealing Hg^2+^ enrichment in the gills, eyes, brain, liver, kidneys, and select muscle regions. Previous studies have reached similar conclusions in other fish species.^31^ Gills and eyes show significant accumulation due to direct contact with the water environment, while the liver, being the main metabolic organ, also accumulates Hg^2+^. Notably, the zebrafish larvae were exposed to a high dose of Hg^2+^ for a short period, simulating acute Hg^2+^ poisoning. Despite the limited ability of Hg^2+^ to penetrate the blood–brain barrier, accumulation is observed in the brains of larvae treated with high doses. These results indicate that Hg^2+^ is mainly present in the head and digestive organs, suggesting these parts should be avoided when consuming fish. Additionally, the gills and eyes could serve as detection sites due to their high accumulation capacity and accessibility to Hg^2+^.

**Figure 6:**
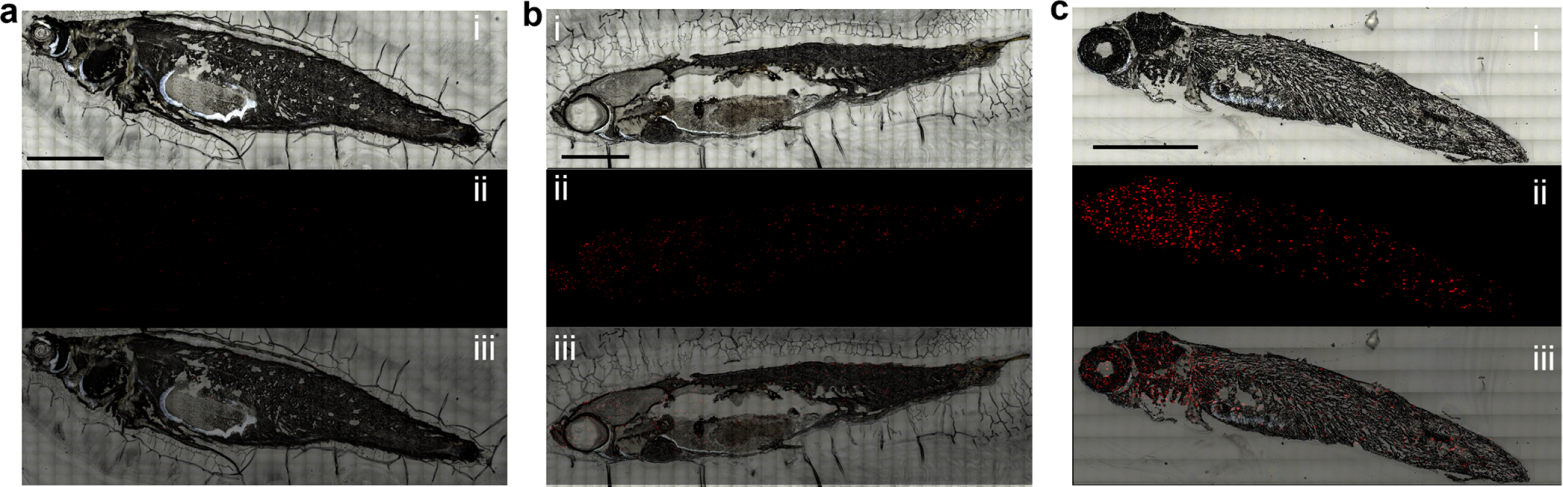
Comparison results of Hg^2+^ zebrafish slices with CF Au/4-MB@Ag NCs (a), blank zebrafish slices with CF Au/4-MB@Ag NCs-aptamer (b), and Hg^2+^ zebrafish slices with CF Au/4-MB@Ag NCs-aptamer (c). (i–iii) The optical images of the zebrafish slices, the mapping results of relative SERS intensities of Hg^2+^ with CF Au/4-MB@Ag NCs-aptamer in the zebrafish slices, and the overlap images of (i) and (ii). All the scale bars are 2000 μm.

#### Real Water and Herbal Sample Analysis

Tap water and herbal plants (Coicis Semen and Vaccariae Semen) were also investigated as real sample matrices. Different concentrations of Hg^2+^ ions were mixed with tap water, Coicis Semen, and Vaccariae Semen, and then SERS detection was performed. Figure S17 shows the typical SERS spectra of Hg^2+^ in herbal plants and tap water. Due to the excellent sensitivity and reliability of our proposed detection system, the quantitative and IS peaks at 1511 and 2222 cm^–1^, respectively, are clearly distinguishable in the samples. Despite the complex matrix of herbal plants reducing the SERS intensity of the characteristic peaks, the IS signal also decreases; thus, the relative intensity of the quantitative peak remains stable compared to the standard solution. To further evaluate detection accuracy, we calculated the concentration of Hg^2+^ in herbal plants based on the relative intensity of the quantitative peaks and compared it to the standard curve in Figure 4, as shown in Table S3. The detected concentrations closely matched the spiked concentrations, with a recovery between 80% and 120%. Notably, the maximum RSD of the calibration signal was only 10.7%, confirming the reliability of the results.

## Conclusion

In summary, this study presents a new aptamer-assisted SERS method for the quantitative detection and *in situ* imaging of heavy metal ions in herbal plants, tap water, and zebrafish. The mechanism that powers this platform thrives on the precise recognition of aptamers, harnessing the innovative capabilities of unique concave-faceted SERS substrates to amplify signals. The proposed CF Au/4-MB@Ag NCs can self-assemble into nanogaps that accommodate aptamers without compromising their structure, significantly enhancing the detection and imaging performance due to the strong localized electromagnetic fields generated by these gaps. The effectiveness of this strategy was evaluated using three heavy metal ions, achieving broader detection ranges, especially for Hg^2+^ and Cd^2+^. Given the large number of aptamers and DNAzymes for various metal ions, this method is poised to be a general metal sensing strategy in a complex sample matrix. The reproducibility of the method was validated by spatially mapping the SERS intensity across up to 600 spectra, yielding an RSD of less than 10%. This finding robustly confirms the toxic accumulation behavior of Hg^2+^ in zebrafish. This work makes a compelling case in the analysis and *in situ* imaging of heavy metal ions by using the innovative SERS method. Even more noteworthy is that it unveils a visionary strategy for an aptamer-based SERS platform, focused on achieving precise detection and sensitive imaging of targets that reveal weak or negligible Raman signals.

## Supplementary Material



## Notes

Additional experimental details, materials, methods, figures, and tables, as mentioned in the text (PDF)
