# Supplementary material for: A Concave Nanogap for Ultrasensitive Aptamer-Based SERS Detection and In Situ Imaging of Heavy Metal Ions
Source: Anal Chem. 2025 Oct 16;97(42):23516–25. doi: 10.1021/acs.analchem.5c04843 (PMC12573227; doi:10.1021/acs.analchem.5c04843)
Supplement: Supplementary file 1 [file ac5c04843_si_001.pdf]

SUPPORTING INFORMATION

A CONCAVE NANOGAP FOR ULTRASENSITIVE APTAMER-BASED SERS DETECTION AND IN SITU IMAGING OF HEAVY METAL IONS

Ting Wang [a†], Feiya Sheng [b†], Sijia Wu [a], Yifei Mao [a], Juewen Liu [c], Peng Li [a, *], Jinchao Wei [a, *]

[a] Macau Centre for Research and Development in Chinese Medicine, State Key Laboratory of Mechanism and Quality of Chinese Medicine, Institute of Chinese Medical Sciences, University of Macau, Macau 999078, China

[b] School of Basic Medical Sciences, Chengdu University, Chengdu 610106, China

[c] Department of Chemistry, Waterloo Institute for Nanotechnology, University of Waterloo, Waterloo, Ontario N2L 3G1, Canada

* Corresponding authors:

E-mail addresses:

pli1978@hotmail.com; pengli@um.edu.mo; (P. LI);

wjc551@hotmail.com; jinchaowei@um.eud.mo; (J.C. WEI);

Chemicals and equipment

Chloroauric acid hydrate ($HAuCl_4 \cdot 4H_2O$, 99.99%), Sodium borohydride ($NaBH_4$, 98%), silver nitrate ($AgNO_3$, 99.85%), sodium hydroxide (NaOH, 97%) and $Cd(NO_3)_2 \cdot 4H_2O$ (99%) were purchased from Sigma-Aldrich (St. Louis, MO, U.S.A.). Ascorbic acid (AA, $C_6H_8O_6$, 99.99%), 4-Mercaptobiphenylcarbonitrile (4-MB, $C_{13}H_9NS$, ≥ 95%) and Zinc nitrate hydrate ($Zn(NO_3)_2 \cdot 6H_2O$, ≥ 99%) were purchased from Aladdin Chemical Co. Ltd, China. Cetyltrimethylammonium chloride (CTAC, $CH_3(CH_2)_{15}N(Cl)(CH_3)_3$, 99%), Mercury nitrate monohydrate ($Hg(NO_3)_2 \cdot H_2O$, 98%) Cobalt nitrate hexahydrate ($Co(NO_3)_2 \cdot 6H_2O$, 99%), Cupric chloride hydrate ($CuCl_2 \cdot 2H_2O$, 99.99%), Chromium(III) nitrate nonahydrate ($Cr(NO_3)_3 \cdot 9H_2O$, 99.95%), Lead chloride ($PbCl_2$, 99.99%), Sodium chloride (NaCl, 99.5%), Potassium chloride (KCl, 99.5%) and Magnesium chloride ($MgCl_2 \cdot 6H_2O$, 99.99%) were acquired by Macklin Biochemical (Shanghai, China). Calcium nitrate tetrahydrate ($Ca(NO_3)_2 \cdot 4H_2O$, ≥ 99%) was purchased from Xilong scientific Co. Ltd, China.

Scanning transmission electron microscopy-energy dispersive X-ray spectroscopy (STEM-EDS) elemental mapping images and transmission electron microscopy (TEM) were obtained with transmission electron microscopy (Talos F200X, FEI, Massachusetts, USA) at an accelerating voltage of 200 kV. Scanning electron microscopy (SEM) images were captured by field emission scanning electron microscopy (FESEM, Zeiss Sigma, Germany). Raman spectra were measured on confocal Raman microscope (InVia, Renishaw, UK). UV-Vis absorption spectral were obtained on a DR 6000 UV-Vis spectrophotometer (HACH, Colorado, USA). The syringe pump was KDS Legato 270 (KD Scientific, Inc., USA).

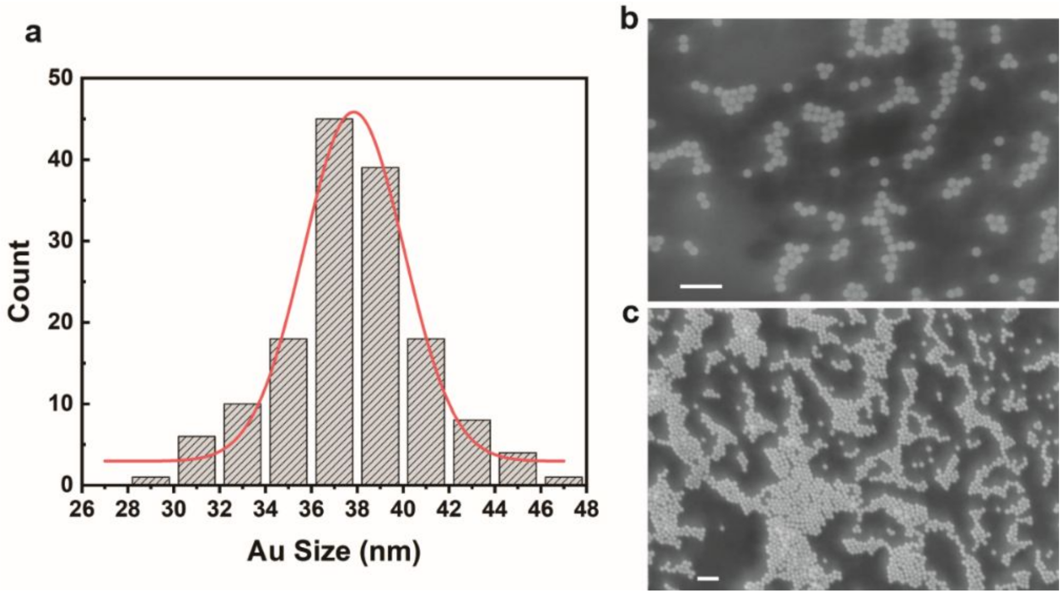

Figure S1. a) Size statistics of the Au NPs. b) and c) Typical SEM images of the Au NPs, and the scale bar is 200 nm.

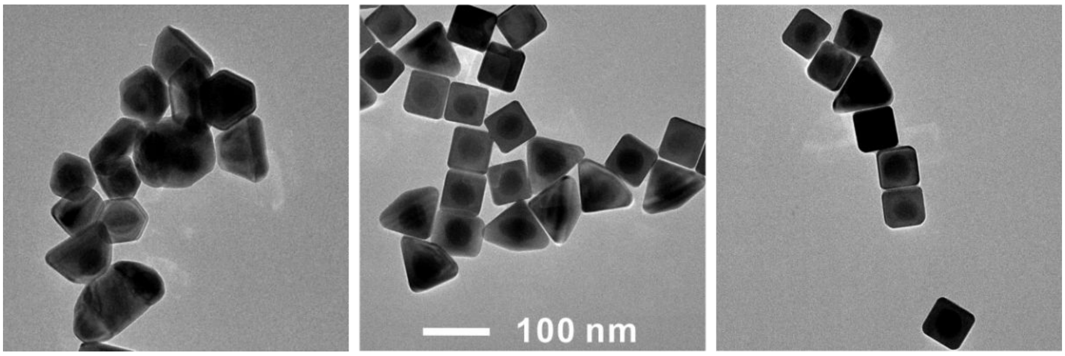

Figure S2. Typical TEM image of Au/4-MB@Ag NPs. The images from left to right correspond to 1×10^{-5} M, 1×10^{-6} M and 1×10^{-7} M 4-MB.

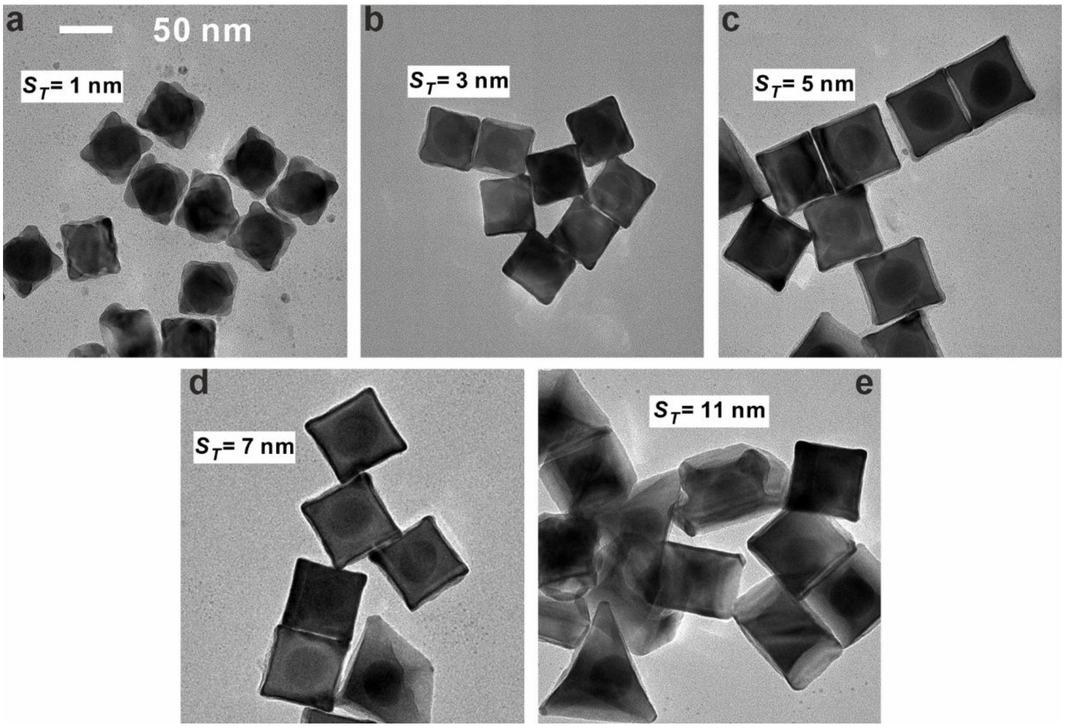

Figure S3. Typical TEM image of CF Au/4-MB@Ag NCs with different shell thickness. The thickness is 1 nm (a), 3 nm (b), 5 nm (c), 7 nm (d), 11 nm (e).

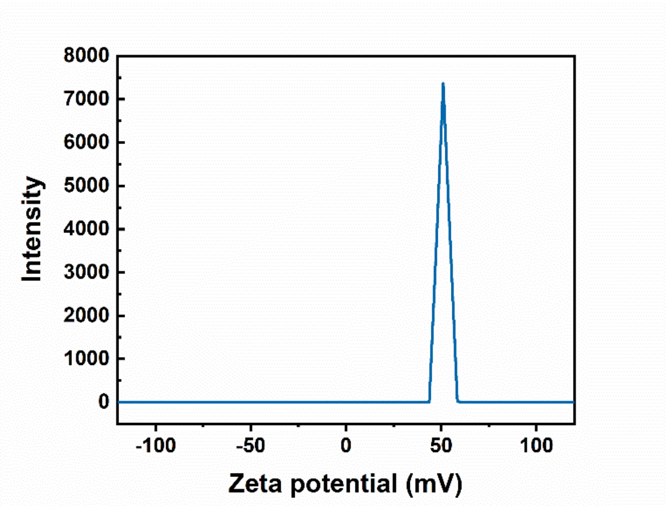

Figure S4. The zeta potential of CF Au/4-MB@Ag NCs.

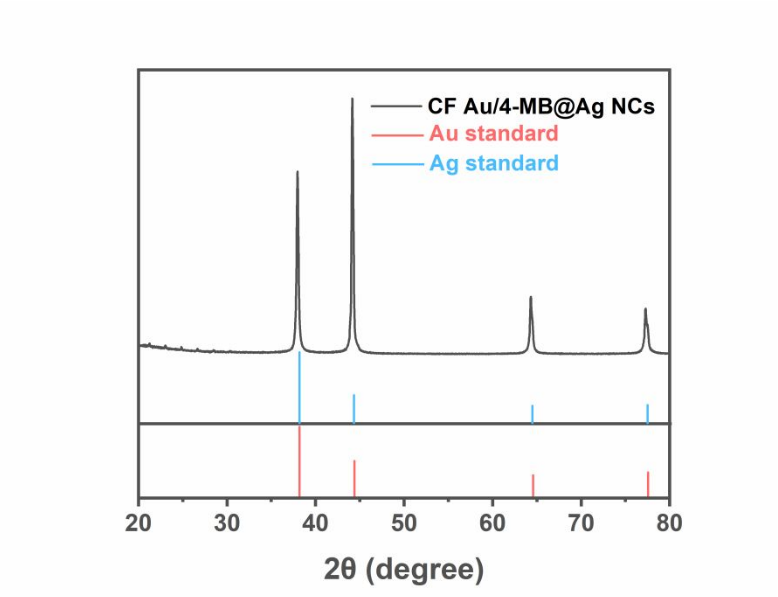

Figure S5. The XRD pattern of CF Au/4-MB@Ag NCs.

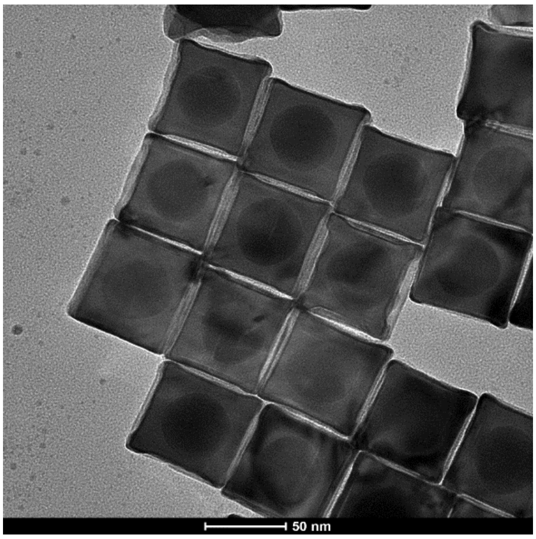

Figure S6. Typical SEM image of ~5nm CF Au/4-MB@Ag NCs.

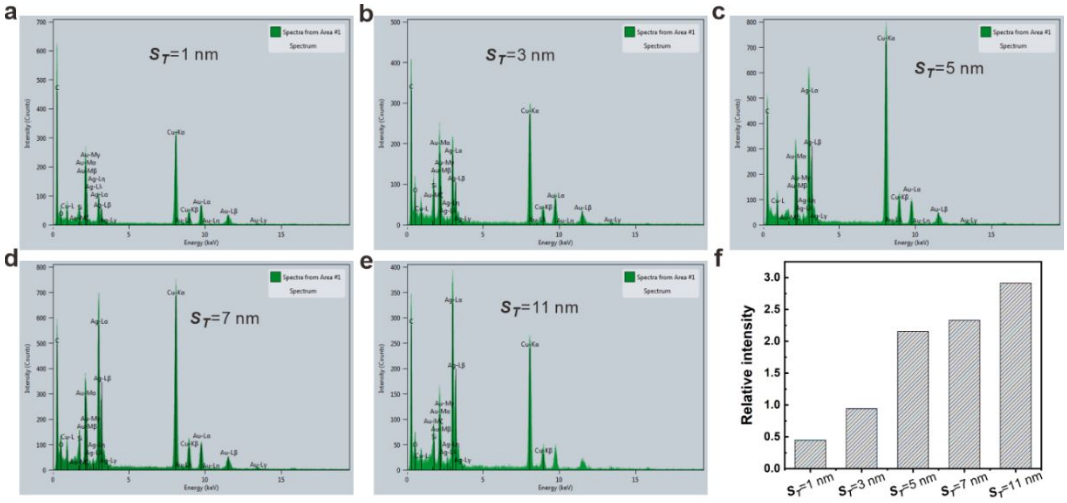

Figure S7. a-e) The EDS spectrum of different shell thickness CF-Au/4-MB@Ag NCs. f) The relative intensity of Ag elements and Au elements.

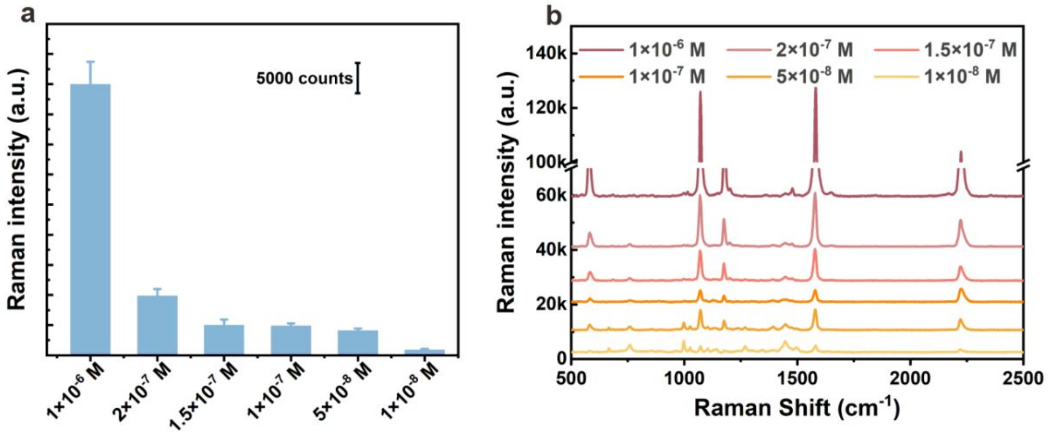

Figure S8. a) The SERS intensity of different 4-MB concentration in ~45nm Au@4-MB@Ag NCs. b) The corresponding typical SERS spectrum of different 4-MB concentration in ~45nm Au@4-MB@Ag NCs.

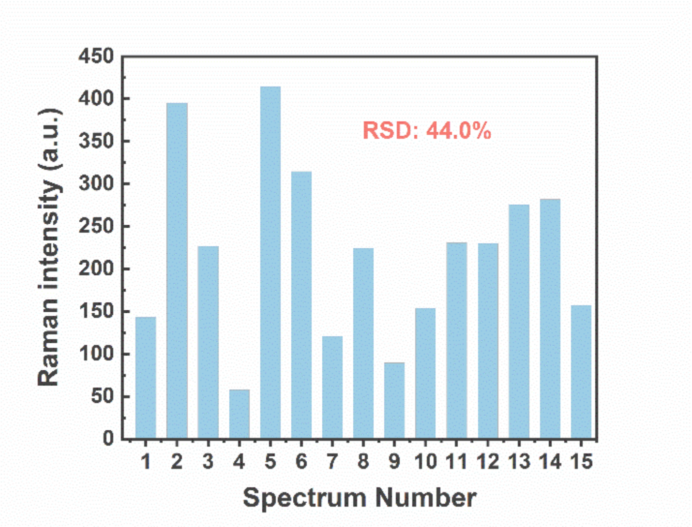

Figure S9. The SERS intensity of IS peak of CF-Au/4-MB@Ag NCs with 5×10^{-9} M 4-MB.

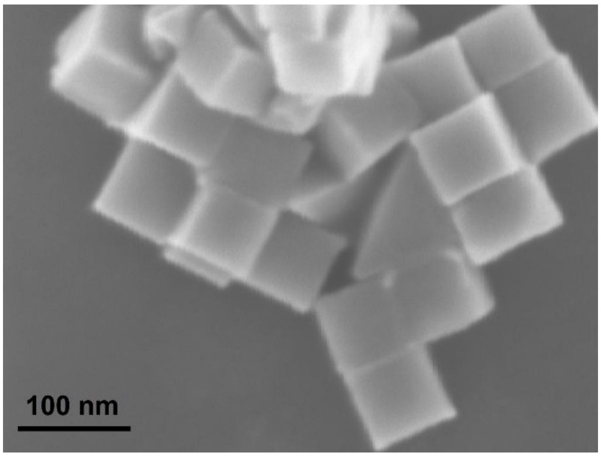

Figure S10. SEM image of CF Au/4-MB@Ag NCs-aptamer.

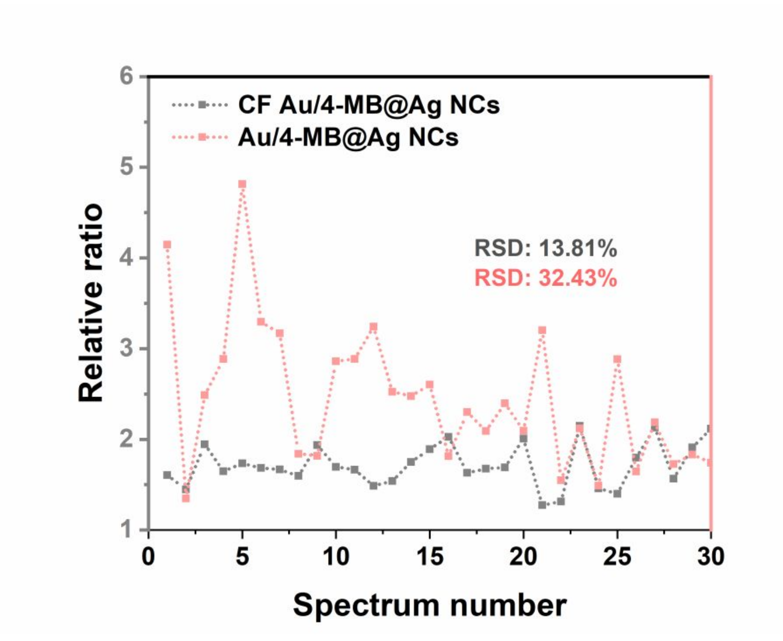

Figure S11. The comparison results of randomly selected 40 points with blank sample of CF Au/4-MB@Ag NCs-aptamer and Au/Au-MB@Ag NCs-aptamer

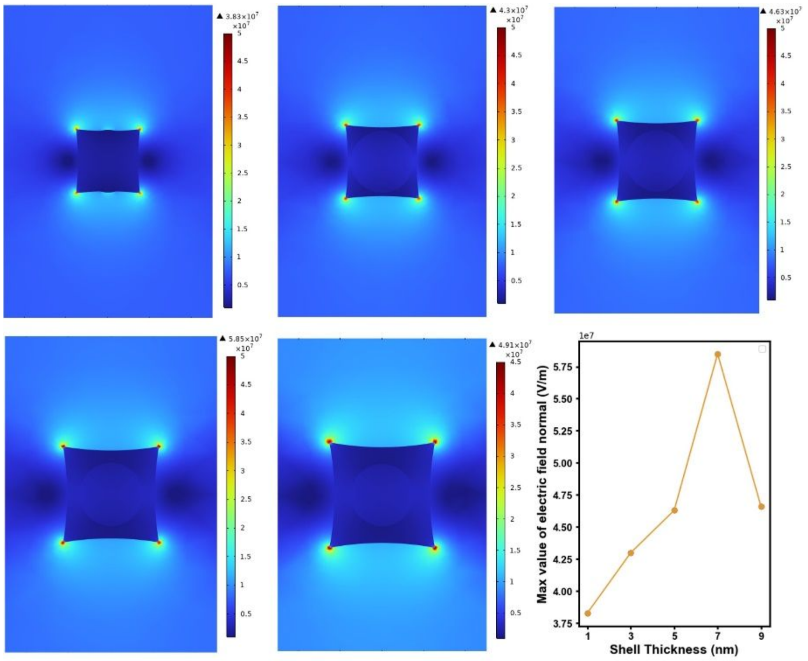

Figure S12. Simulation of localized electric field (V/m) distribution of different thickness shell of CF Au/4-MB@Ag NCs, a) 0 nm, b) 3 nm, c) 5 nm, d) 7 nm, e) 9 nm. (f) the maximum value of electric field normal in different thickness shell CF Au/4-MB@Ag NCs.

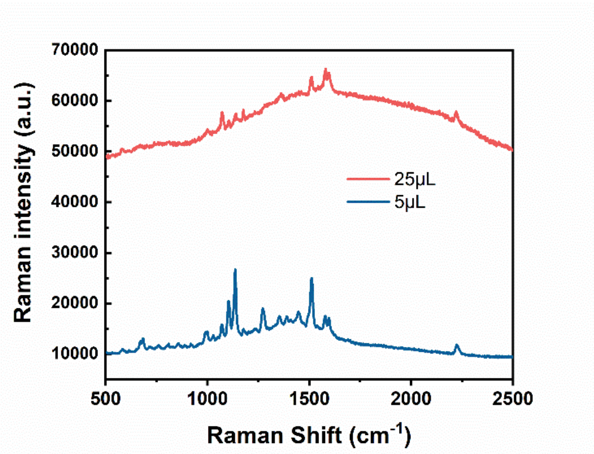

Figure S13. SERS spectrum of CF-Au/4-MB@Ag NCs with 5 μL and 25 μL Hg^{2+}-aptamer (with baseline).

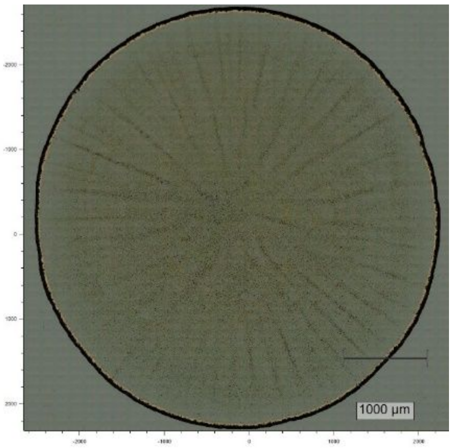

Figure S14. The optical images of the CF-Au/4-MB@Ag NCs-Aptamer distributed on the silicon wafer.

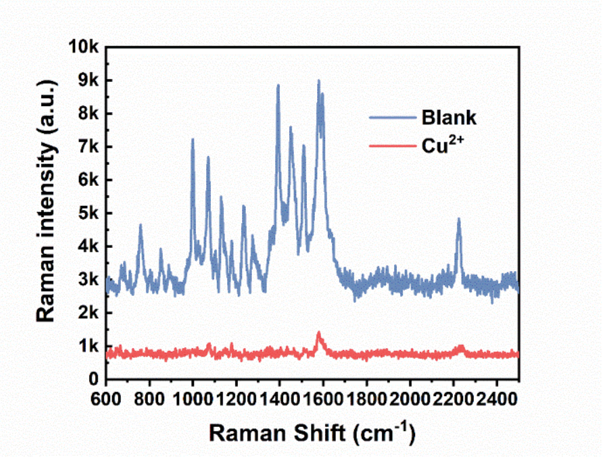

Figure S15. The SERS spectrum of CF-Au/4-MB@Ag NCs-Aptamer in ultrapure water and 10 μM Cu^{2+} solution.

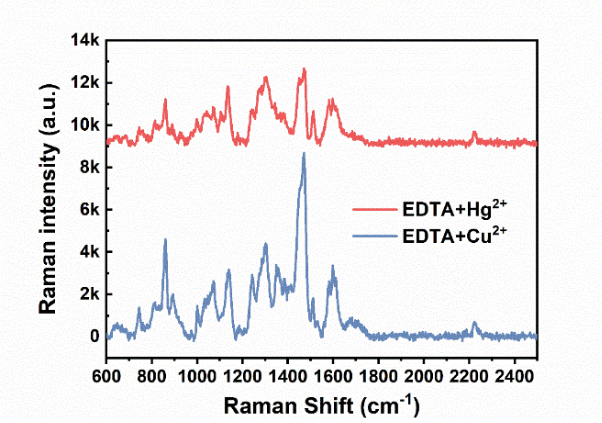

Figure S16. The SERS spectrum of CF Au/4-MB@Ag NCs-Aptamer with EDTA and Hg^{2+} and Cu^{2+}.

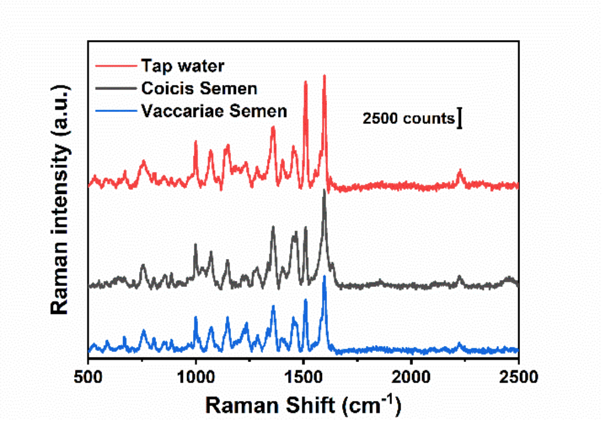

Figure S17. The typical SERS spectra of Hg^{2+} in herbal plants and tap water.

Table S1. Comparison of mapping area and corresponding results

No.	Substrate	IS	Area (μm^2)	Number of spectra	RSD (%)	Reference
1	MoS_2@AuNSs	4-MBA	/	10	1.57%	(Zhou et al., 2023)
2	Au@4-MBN@Ag@LMG@ZIF-8	4-MBN	/	10	4.91%	(Sun et al., 2024)
3	Au@MGITC@SiO_2	MGITC	/	20	3.95%	(Fu et al., 2022)
4	Au NPs array	Acetone	/	40	5.9%	(Yu et al., 2018)
5	Au nanorods	chloroform	/	50	6.2%	(Tian et al., 2018)
6	Chiral Au nanocrystals	MB	/	50	9%	(Tian et al., 2024)
7	Semi-wrapped Au@PB	PB	40×40	256	8.55%	(Wang et al., 2023)
8	Au@4MBN@SiO_2	4MBN	40×40	400	10.68%	(Chen et al., 2025)
9	NaCMC-Si/PMMA@MoS_2@Ag hybrid substrate	Si NPs	1000×1000	2500	11.6%	(Jiang et al., 2022)
10	CF Au@4-MB@Ag NCs	4MB	Whole circle edge	611	9.8%	This work

Table S2. The comparison of LOD of heavy metal ions in different core-shell SERS substrate

No.	substrate	Heavy metal ions	Linear range	LOD	reference
1	Au@gap@AuAg NRs	Hg^{2+}	0.005 — 1.0 ng/mL	0.001 ng/mL	(Yuan et al., 2019)
2	SiO_2@Au NPs	Hg^{2+}	10 nM — 10 μM	10.0 nM	(Lu et al., 2018)
3	PTh-MnS/CoS NPs	Hg^{2+}	1 nM — 100 μM	1.0 nM	(Tefery et al., 2025)
4	Au@Ag NPs	Hg^{2+}	1×10^{-3} — 1×10^{2} μg/g	10^{-3} μg/g	(Hassan et al., 2021)
5	Au@PS MPs	Hg^{2+}	1 nM — 10 μM	0.1 nM	(Zou et al., 2019)
6		Pb^{2+}	10 nM — 100 μM	1.0 nM	
7	Au@Ag NRs	Pb^{2+}	0.5 — 1000 μg/L	0.021 μg/L	(Liu et al., 2024)
8	SiO_2@Au NPs	Cd^{2+}	0.1 — 2 ppm	0.1 ppm	(Thatai et al., 2015)
9	CF Au@4-MB@Ag NCs	Hg^{2+}	0.1 — 1000 nM	0.1 nM	This work

	Pb^{2+}	0.1 — 100 nM	0.1 nM
	Cd^{2+}	10 — 10000 nM	10 nM

Table S3. Detection of Hg^{2+} in herbal plants and tap water

Analyte	Matrices	Spiked (nM)	Detected (nM)	Recovery (%)	RSD (%, *n*=3)
**Hg^{2+}**	Coicis Semen	500	452	90	2.61
		100	113	113	2.63
		1	1.13	113	3.51
	Vaccariae Semen	500	487	97	1.92
		100	117	117	1.81
		1	1.15	115	3.50
	Tap water	500	443	88	6.57
		100	107	107	3.60
		1	1.09	109	10.69

Reference

(1) Chen, Z.; Sun, N.; Li, J.; Zheng, J.; Wang, Y.; Zhou, X.; Zheng, B. SERS Calibration Substrate with a Silent Region Internal Standard for Reliable Simultaneous Detection of Multiple Antibiotics in Water. *Talanta* **2025**, *283*, 127133. https://doi.org/10.1016/j.talanta.2024.127133.

(2) Fu, B.; Tian, X.; Song, J.; Wen, B.; Zhang, Y.; Fang, P.; Li, J. Self-Calibration 3D Hybrid SERS Substrate and Its Application in Quantitative Analysis. *Anal. Chem.* **2022**, *94* (27), 9578–9585. https://doi.org/10.1021/acs.analchem.2c00436.

(3) Hassan, M. M.; Ahmad, W.; Zareef, M.; Rong, Y.; Xu, Y.; Jiao, T.; He, P.; Li, H.; Chen, Q. Rapid Detection of Mercury in Food via Rhodamine 6G Signal Using Surface-Enhanced Raman Scattering Coupled Multivariate Calibration. *Food Chem.* **2021**, *358*, 129844. https://doi.org/10.1016/j.foodchem.2021.129844.

(4) Jiang, J.; Xu, L.; Zhang, Y.; Ma, J.; Gu, C.; Zhou, X.; Wei, G.; Jiang, T. Quantitative and Recyclable SERS Detection Induced by Tunable Raman Internal Standard from Embedded Silicon Nanoparticles. *Sens. Actuators, B* **2022**, *366*, 131989. https://doi.org/10.1016/j.snb.2022.131989.

(5) Liu, M.; Zareef, M.; Zhu, A.; Wei, W.; Li, H.; Chen, Q. SERS-Based Au@Ag Core-Shell Nanoprobe Aggregates for Rapid and Facile Detection of Lead Ions. *Food Control* **2024**, *155*, 110078. https://doi.org/10.1016/j.foodcont.2023.110078.

(6) Lu, Y.; Zhong, J.; Yao, G.; Huang, Q. A Label-Free SERS Approach to Quantitative and Selective Detection of Mercury (II) Based on DNA Aptamer-Modified SiO2@Au Core/Shell Nanoparticles. *Sens. Actuators, B* **2018**, *258*, 365–372. https://doi.org/10.1016/j.snb.2017.11.110.

(7) Sun, Y.; Zhang, Y.; Ren, H.; Qiu, H.; Zhang, S.; Lu, Q.; Hu, Y. Highly Sensitive SERS Sensors for Glucose Detection Based on enzyme@MOFs and Ratiometric Raman. *Talanta* **2024**, *271*, 125647. https://doi.org/10.1016/j.talanta.2024.125647.

(8) Tefery, T.; Jayachitra, R.; Prasannan, A.; Damastuti, R.; Tsai, H. Tailoring polythiophene nanocomposites with MnS/CoS nanoparticles for enhanced SERS detection of mercury ions in water. *Colloid Surface A* **2025**, *705*, 135715. https://doi.org/10.1016/j.talanta.2024.125647.

(9) Thatai, S.; Khurana, P.; Prasad, S.; Kumar, D. Plasmonic Detection of Cd^{2+} Ions Using Surface-Enhanced Raman Scattering Active Core–Shell Nanocomposite. *Talanta* **2015**, *134*, 568–575. https://doi.org/10.1016/j.talanta.2014.11.024.

(10) Tian, L.; Su, M.; Yu, F.; Xu, Y.; Li, X.; Li, L.; Liu, H.; Tan, W. Liquid-State Quantitative SERS Analyzer on Self-Ordered Metal Liquid-like Plasmonic Arrays. *Nat. Commun.* **2018**, *9* (1), 3642. https://doi.org/10.1038/s41467-018-05920-z.

(11) Tian, Y.; Wu, F.; Lv, X.; Luan, X.; Li, F.; Xu, G.; Niu, W. Enantioselective Surface-Enhanced Raman Scattering by Chiral Au Nanocrystals with Finely Modulated Chiral Fields and Internal Standards. *Adv. Mater.* **2024**, *36* (35), 2403373.

https://doi.org/10.1016/j.colsurfa.2024.135715.

(12) Wang, T.; Ji, B.; Cheng, Z.; Chen, L.; Luo, M.; Wei, J.; Wang, Y.; Zou, L.; Liang, Y.; Zhou, B.; Li, P. Semi-Wrapped Gold Nanoparticles for Surface-Enhanced Raman Scattering Detection. *Biosens. Bioelectron.* **2023**, *228*, 115191. https://doi.org/10.1016/j.bios.2023.115191.

(13) Yu, F.; Su, M.; Tian, L.; Wang, H.; Liu, H. Organic Solvent as Internal Standards for Quantitative and High-Throughput Liquid Interfacial SERS Analysis in Complex Media. *Anal. Chem.* **2018**, *90* (8), 5232–5238.

https://doi.org/10.1021/acs.analchem.8b00008.
(14) Yuan, A.; Wu, X.; Li, X.; Hao, C.; Xu, C.; Kuang, H. Au@gap@AuAg Nanorod Side-by-Side Assemblies for Ultrasensitive SERS Detection of Mercury and Its Transformation. *Small* **2019**, *15* (27), 1901958. https://doi.org/10.1002/smll.201901958.
(15) Zhou, P.; Cheng, S.; li, Q.; Pang, Y.; Xiao, R. Multifunctional MoS2@AuNSs Nanoflakes as SERS and Photothermal Tags for Single-Cell Bacterial Detection and in-Situ Inactivation. *Chem. Eng. J.* **2023**, *471*, 144514. https://doi.org/10.1016/j.cej.2023.144514.
(16) Zou, Q.; Li, X.; Xue, T.; Zheng, J.; Su, Q. SERS Detection of Mercury (II)/Lead (II): A New Class of DNA Logic Gates. *Talanta* **2019**, *195*, 497–505. https://doi.org/10.1016/j.talanta.2018.11.089
